# The effect of dams and seasons on malaria incidence and anopheles abundance in Ethiopia

**DOI:** 10.1186/1471-2334-13-161

**Published:** 2013-04-03

**Authors:** Delenasaw Yewhalaw, Yehenew Getachew, Kora Tushune, Kifle W/Michael, Wondwossen Kassahun, Luc Duchateau, Niko Speybroeck

**Affiliations:** 1 Department of Biology, College of Natural Sciences, Jimma University, Jimma, Ethiopia; 2 Department of Horticulture & Plant Science, College of Agriculture & Veterinary Medicine, Jimma University, Jimma, Ethiopia; 3 Department of Health Service Management, College of Public Health & Medical Sciences, Jimma University, Jimma, Ethiopia; 4 Department of Epidemiology and Biostatistics, College of Public Health & Medical Sciences, Jimma University, Jimma, Ethiopia; 5 Department of Comparative Physiology and Biometrics, Faculty of Veterinary Medicine, Ghent University, Gent, Belgium; 6 Institute of Health and Society, Faculty of Public Health, Université Catholique de Louvain, Brussels, Belgium

**Keywords:** Malaria incidence, *P. falciparum*, Mosquito, Dam, Season, Ethiopia

## Abstract

**Background:**

Reservoirs created by damming rivers are often believed to increase malaria incidence risk and/or stretch the period of malaria transmission. In this paper, we report the effects of a mega hydropower dam on *P. falciparum* malaria incidence in Ethiopia.

**Methods:**

A longitudinal cohort study was conducted over a period of 2 years to determine *Plasmodium falciparum* malaria incidence among children less than 10 years of age living near a mega hydropower dam in Ethiopia. A total of 2080 children from 16 villages located at different distances from a hydropower dam were followed up from 2008 to 2010 using active detection of cases based on weekly house to house visits. Of this cohort of children, 951 (48.09%) were females and 1059 (51.91%) were males, with a median age of 5 years. Malaria vectors were simultaneously surveyed in all the 16 study villages. Frailty models were used to explore associations between time-to-malaria and potential risk factors, whereas, mixed-effects Poisson regression models were used to assess the effect of different covariates on anopheline abundance.

**Results:**

Overall, 548 (26.86%) children experienced at least one clinical malaria episode during the follow up period with mean incidence rate of 14.26 cases/1000 child-months at risk (95% CI: 12.16 - 16.36). *P. falciparum* malaria incidence showed no statistically significant association with distance from the dam reservoir (*p* = 0.32). However, *P. falciparum* incidence varied significantly between seasons (*p* < 0.01). The malaria vector, *Anopheles arabiensis*, was however more abundant in villages nearer to the dam reservoir.

**Conclusions:**

*P. falciparum* malaria incidence dynamics were more influenced by seasonal drivers than by the dam reservoir itself. The findings could have implications in timing optimal malaria control interventions and in developing an early warning system in Ethiopia.

## Background

Globally, 60% of the world’s large river systems are impacted by dams [1]. Such dams have a wide range of benefits, mainly of economic nature [2-4]. On the other hand, such dams can have profound effects on the survival, density and distribution of disease vectors and parasites such as malaria, by altering the local ecology and habitats. The altered vector/parasite ecology modifies the transmission of vector-borne diseases and subsequently the local disease incidence and prevalence [5-8]. Worldwide, 18.9 million people living close to large dams are at risk of malaria. In Africa alone, 9.4 million people live near to large dams [9].

Ethiopia has recently constructed a large number of dams to produce electricity, irrigate agricultural lands, control flood, reduce poverty and sustain economic growth. Examples of mega hydropower projects are Gilgel-Gibe I and II (completed), Gibe III and the Grand Ethiopian Renaissance Dam (under construction) with a capacity of generating 184MW, 420MW, 1870MW and 6000MW, respectively. More damming projects are underway, as Ethiopia currently uses only 3% of its hydroelectric potential [10].

In Ethiopia, little or no information is available on the effect of such large hydropower dams on *P. falciparum* malaria incidence. This study is the first longitudinal study conducted in Ethiopia to investigate the effect of large hydropower dams and on malaria incidence risk. The findings of this study could assist the development of a dam-associated malaria control programme.

Thus, the objective of this longitudinal study was: 

1. To investigate whether the distance from the dam reservoir has an influence on *P. falciparum* malaria incidence and/or on malaria vector abundance, and

2. To investigate the dynamics of malaria and its vectors with season.

## Methods

### Study area and population

**Figure 1:**
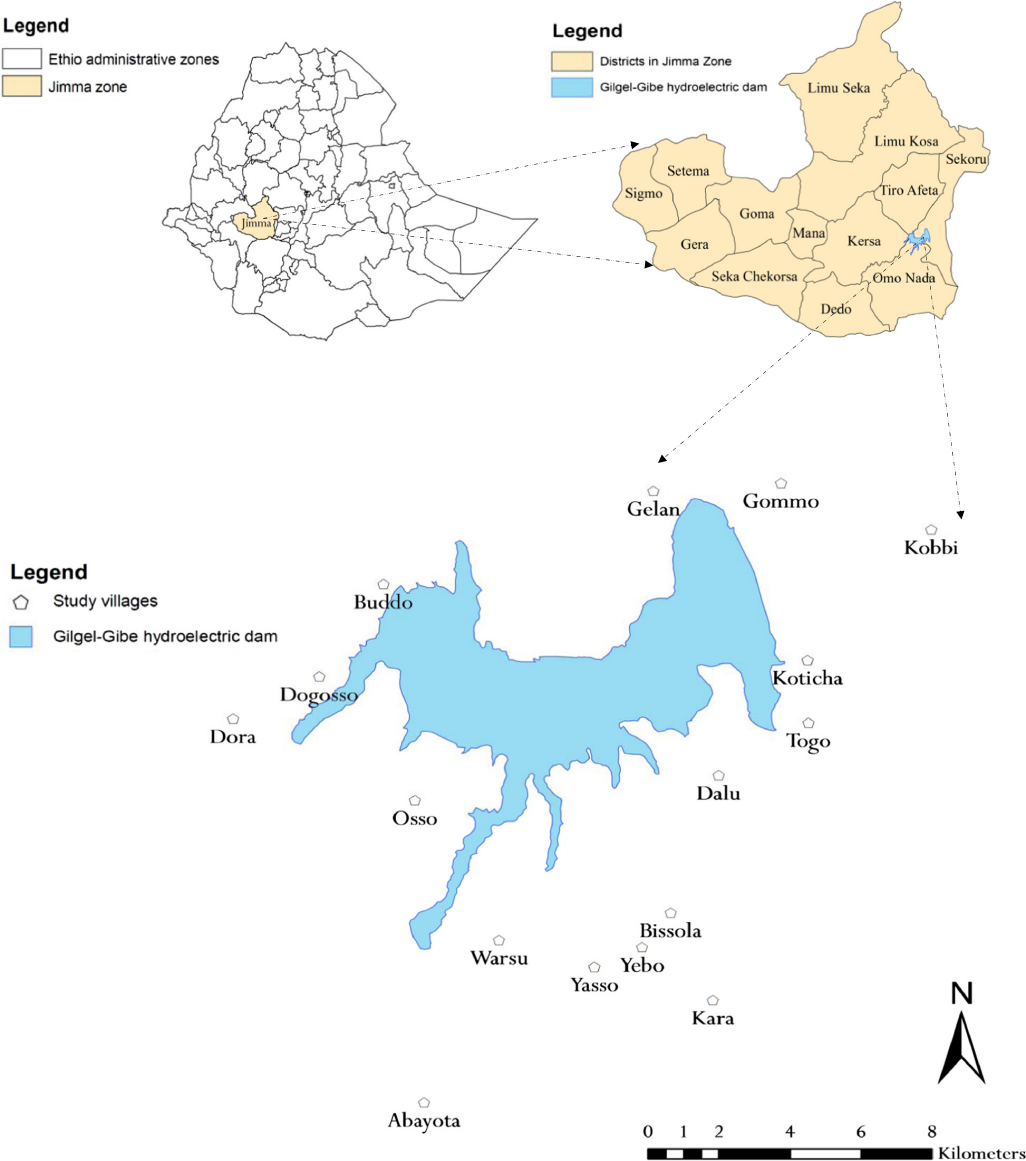
Map of Ethiopia showing districts in Jimma zone, Gilgel-Gibe hydroelectric dam and study villages.

A longitudinal cohort study was conducted over a period of two years (July 2008 - June 2010) among children less than 10 years living in 16 study villages around the Gilgel-Gibe hydropower reservoir in south-western Ethiopia (Figure 1). Details of the study setting are described elsewhere [11,12]. In brief, prior to the study, all villages within 10 km radius (265 - 9046 meters) from the dam reservoir shore were first identified and then 16 villages, located at different distances from the dam reservoir shore, were randomly selected among villages starting from the closest villages to the farthest ones based on similar eco-topography, access to health facilities, without major impounded water nearby and homogeneous with respect to socio-cultural and daily economic activities.

### Sample size and participant selection

The study was designed to detect a 5% difference in malaria incidence rate and the sample size calculation (with *α* = 0.05 and *β* = 0.20) was based on a foreseen *P. falciparum* malaria incidence rate of 15% and 10% in ’distant’ and ’nearby’ villages, respectively. A dropout rate of 10% and a design factor equal to three (to adjust for the clustering effect) were used to get a representative sample size of 2080 study participants. Hence, 130 children were randomly selected from a list of all children less than 10 years of age in each of the 16 villages for the follow-up study.

### Clinical *Plasmodiumfalciparum* malaria follow-up and laboratory processing

The cohort of 2080 children was monitored longitudinally for *P. falciparum* malaria incidence by active case detection. Children residing in the study area for at least six months were eligible for the study. Site-based data collectors with at least secondary school education were recruited, trained and assigned to collect data in each of the 16 study villages. They were trained in recording body temperature, making thick and thin blood smears and administration of anti-malarial drugs. Each selected child was followed-up by trained data collectors at weekly interval based on house to house visits. If the child presented itself with fever, an axillary body temperature ≥ 37.5°C or when the mother or guardian reported that the child had fever in the past 24 hours, a finger prick blood sample was taken. Blood smears (thick and thin) were stained with Giemsa stain following a standard technique [13] and were read by experienced investigators on site. A film was recorded as negative, if two hundred optical fields under 1000x oil immersion magnification were all negative. Films positive for plasmodia parasites and 10% of sample films negative for parasites were read by independent blinded senior investigators at Jimma University hospital laboratory for confirmation. All subjects with documented fever got treatment on site as per the national malaria diagnosis and treatment guidelines. Whenever necessary mothers or guardians were advised to seek further treatment at the nearby health center.

### Ethical approval

The study was reviewed by the World Health Organization/TDR research ethics committee and ethical approval was obtained from the research and ethics committee of Jimma University. Verbal and written signed informed consent was obtained from the mother or caregiver of each child before enrollment of the child in the study.

### Mosquito sampling and identification

Adult mosquitoes were collected monthly in all study villages to assess the effect of distance from the dam reservoir and season on mosquito density. Two houses, one located at the center and one at the periphery of the village were selected for mosquito collection in each of the 16 study villages. Mosquitoes were collected one day a month, from 1800 to 0600 hours from each of the two selected houses using light trap catches. Traps were hung from the roof supports or pillars at the foot end of the bed or traditional mud-made sleeping place. The trap was suspended about 1.5 meters above the bed. In all the selected houses occupants were provided with new untreated nets prior to the start of the entomological survey. Collection bags were retrieved from traps in the morning from 0800 to 0900 hours. The collected mosquitoes were then transferred into paper cups, killed using chloroform, sorted by genus and sex and then counted. The number of human occupants and potential hosts in each surveyed house during the previous night was recorded. Space spray catches (SSCs) were not included as the mosquito population in the study area showed high levels of insecticide resistance [11,12]. Morphological identification of collected mosquitoes was carried out using standard keys [14]. Morphologically identified mosquitoes were then scored as unfed, fed, half gravid and gravid. Members of the *An. gambiae* s.l complex were further assigned to sibling species using polymerase chain reaction (PCR) techniques as described by Hunt et al [15] and modified by Yewhalaw et al [16].

### Climatological data

Monthly rainfall (mm), relative humidity (%), maximum and minimum temperature (°C) of the study area were obtained from the south-western branch regional office of the Ethiopian Meteorological Agency for the two years of the follow-up study. Similar climatic conditions were assumed for all the sixteen villages.

## Data analysis

*P. falciparum* malaria incidence rates were expressed as the number of cases per 1000 child-months at risk. Two or more consecutive episodes occurring within 30 days of the first episode were considered recrudescent infections and treated as a single episode. The 95% confidence intervals of malaria incidence rates were calculated assuming that the number of new malaria cases is Poisson distributed.

The *P. falciparum* malaria risk was analysed through a time-to-event model, which is the most efficient way to model time-to-event data [17]. A piecewise constant hazard function was used to take into account hazard rate changes between different seasons and years. First, univariable hazard models were fitted to evaluate the marginal effects of different covariates on time-to-malaria. For the multivariable analyses, different covariates were entered into the model simultaneously.

The covariates used were: distance from the dam reservoir shore, season, sex, age, year, and mosquito density. Since children are clustered within villages, village was introduced in the piecewise constant hazard model as a gamma distributed random effect (frailty) to accommodate for the correlations in the data. The data were analyzed using STATA 10 [18].

A mixed-effects Poisson regression model was used to explore the association between mosquito abundance and the following covariates: distance from the dam reservoir shore, year, climatic variables, and season. Village was treated as a random effect in the model. To get better insights in the specific role of the climatic variables, the relation between mosquito density and climatic variables at different lag periods was tested as well. The analysis was performed using PROC GENMOD [19].

## Results

Of 2080 children enrolled at the start of the study (July, 2008), 29 (1.4%) died due to various reasons and 15 (0.72%) migrated elsewhere. Of the migrant children, two children were tracked back and 13 were lost to follow up (until June 2010). Therefore, the effective number of children followed up was 2040 (97.98%) resulting in 48,960 person-months at risk. Of this cohort, 951 children (48.09%) were females and 1059 (51.91%) males. The median age of children in the cohort was five years. In total, 548 (26.86%) children experienced at least one clinical malaria episode during the follow up. The number of children with 1, 2 and 3 clinical malaria episodes were 421 (20.64%), 119 (5.83%) and 7 (0.34%), respectively. One child had five (0.26%) clinical malaria episodes over the two years follow up period. Overall, the number of new *P. falciparum* malaria clinical episodes recorded was 685 resulting in an overall incidence rate of 14 cases/1000 child-months at risk (95% CI: 12.16 - 16.36) and the annual parasite index (API) for year 1 and year 2 was 177/1000 children and 158/1000 children, respectively.

**Figure 2:**
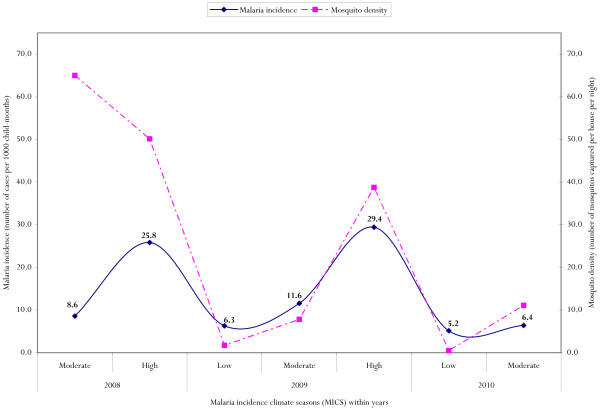
Seasonal dynamics of malaria incidence (number of cases per 1000 child-months) and mosquito density (number of mosquitos captured per house per night) between 2008 and 2010 at the Gilgel-Gibe hydroelectric dam in the Jimma zone, Ethiopia.

Figure 2 presents the seasonal dynamics of *P. falciparum* malaria incidence in the study area over the 2 years study period. Three malaria incidence climate seasons (MICS) could be identified consistently over the two years follow up: a high malaria incidence climate season (HMICS) from August to November, a moderate malaria incidence climate season (MMICS) from April to July, and a low malaria incidence climate season (LMCIS) from December to March. Malaria peaked in October/November following the main rains (25.8 vs. 29.4 cases per 1000 child-months at risk). In general, malaria in the study area also showed marked seasonality as more than 75% of clinical cases occurred in less than 6 months over the two years study period. Figure 2 also indicates the relationship between mosquito abundance and malaria incidence.

Table 1 shows the associations between the hazard of a malaria event and the different covariates. The first column shows the results of the univariable analyses while the second column shows results of the multivariable model with all covariates included. In columns 3 and 4, the mosquito density and the season were respectively dropped from the multivariable model as these two covariates were highly correlated. No significant differences between the first and second year of follow up in the *P. falciparum* risk (362 or 7.39 cases/1000 child-months at risk in the first year and 323 cases or 6.60 cases/1000 child-months at risk, in the second year) were found. No association between *P. falciparum* malaria incidence and the distance from the dam reservoir shore was found. The difference in malaria risk between the two sexes was not significant. However, the difference in malaria risk among the three age strata was significant with children above three years of age having significantly higher *P. falciparum* risk than children less than 3 years of age. A significant association was also observed between *P. falciparum* malaria risk and season (*p* < 0.001) (Table 1).

**Table 1:** Parameter estimates with 95% confidence interval (in parentheses) for the time-to-first malaria based on different univariable and multivariable shared frailty models

**Covariates**		**Univariable model**	**Multivariable models**		
		**HR**^**‡ **^**(95% CI)**	**Adjusted HR (95% CI)**		
Distance		0.989(0.894, 1.093)	0.990(0.896, 1.093)	0.995(0.900, 1.100)	0.993(0.899,1.096)
Sex	Female(ref)	1.000	1.000	1.000	1.000
	Male	0.951(0.803, 1.127)	0.952(0.804, 1.127)	0.952(0.804, 1.128)	0.952(0.804, 1.127)
Age	≤ 3 years(ref)	1.000	1.000	1.000	1.000
	3 to 7 years	1.663 ^∗^(1.117, 2.474)	1.804 ^∗∗^(1.211, 2.687)	1.678 ^∗^(1.126, 2.500)	1.802 ^∗∗^(1.210, 2.685)
	≥ 7 years	1.656 ^∗^(1.098, 2.498)	1.791 ^∗∗^(1.182, 2.713)	1.686 ^∗^(1.112, 2.554)	1.790 ^∗∗^(1.182, 2.712)
Year	Year 1(ref)	1.000	1.000	1.000	1.000
	Year 2	0.991(0.837, 1.1756)	0.921(0.773, 1.097)	0.983(0.826, 1.170)	0.933(0.785, 1.110)
Mosquito density		1.006 ^∗^(1.001, 1.011)	0.997(0.992, 1.003)	1.006 ^∗^(1.001, 1.011)	
Season	Dry season(ref)	1.000	1.000		1.000
	Long rainy season	4.415 ^∗∗^(3.390, 5.750)	4.545 ^∗∗^(3.478, 5.938)		4.455 ^∗∗^(3.421, 5.802)
	Short rainy season	2.182 ^∗∗^(1.580, 3.013)	2.178 ^∗∗^(1.576, 3.008)		2.174 ^∗∗^(1.574, 3.004)

^∗^Significant at *P* < 0.05, ^∗∗^ Significant at *P* < 0.01.

^*†*^Hazard Ratio (HR): is an expression of the hazard or chance of infected with malaria in a particular category as a ratio of the hazard of the events occurring in the reference category. i.e. Estimates of hazard ratios correspond to the hazard of contracting malaria for a child divided by the hazard for a child in the reference category.

Overall, 2353 adult female anopheline mosquitoes belonging to 10 species were collected in 2 years study period. Morphological identification showed that *An. gambiae* s.l., was the most predominant (88%) followed by *An. demelloni* (6.25%) and *An. coustani* (2.68%). All other anopheline species constituted 3.2% of all the collected anopheline species including *An. funestus* and *An. pharoensis*, which have a secondary role in malaria transmission in Ethiopia. Molecular identification of *An. gambiae* complex revealed that over 98.5% of the assayed specimens were *An. arabiensis*.

Table 2 shows the results of mixed-effects Poisson regression model, indicating the association between mosquito density (*An. arabiensis*) and different covariates. The first column shows the results of the univariable analyses while the second column shows results of the multivariable model with all covariates included. In column 3, the climatic variables were dropped from the multivariable model as they were highly correlated with season. There was a significant association between mosquito density and distance from the dam reservoir (*p* < 0.01). Mosquito density decreased by 53% at 6-7 km from the dam, compared with localities close to the dam reservoir. The strong effect of season was modified through the climatic variables indicating that a strong part of the seasonal effect was based on climate. Table 3 shows the relationship between mosquito density and climatic variables at different lag periods. All climatic variables (rainfall, relative humidity and temperature) were strong predictors of mosquito density.

**Table 2:** Parameter estimates with 95% confidence interval (in parentheses) for the association of mosquito density (per trap/house) with different covariates based on univariable and multivariable Poisson regression models

**Covariates**		**Univariable model**	**Multivariable models**	
		**IRR**^**‡ **^**(95% CI)**	**Adjusted IRR (95% CI)**	
Distance		0.721 ^∗∗^(0.581, 0.894)	0.779 ^∗^(0.626, 0.969)	0.734 ^∗∗^(0.585, 0.920)
Year	Year 1(ref)	1.000	1.000	1.000
	Year 2	0.486 ^∗^(0.266, 0.886)	0.704(0.373, 1.327)	0.761(0.410, 1.415)
Climatic variables	Rainfall	1.550 ^∗∗^(0.443, 1.665)	1.091(0.975, 1.222)	
	Relative humidity	1.095 ^∗∗^(1.069, 1.122)	1.046(0.982, 1.114)	
	Maximum temperature	0.580 ^∗∗^(0.474, 0.70)	1.102(0.651, 1.865)	
	Minimum temperature	1.525 ^∗∗^(1.320, 1.761)	0.940(0.827, 1.067)	
Season	Dry season(ref)	1.000	1.000	1.000
	Long rainy season	27.121 ^∗∗^(12.238, 60.106)	9.020 ^∗∗^(3.235, 25.147)	21.048 ^∗∗^(8.549, 51.821)
	Short rainy season	5.508 ^∗∗^(2.802, 10.829)	2.484 ^∗^(1.087, 5.675)	3.720 ^∗∗^(1.789, 7.733)

^∗^Significant at *P* < 0.05, ^∗∗^ Significant at *P* < 0.01.

^*†*^Incidence Rate Ratio (IRR): is obtained by exponentiating the Poisson regression coefficient and it is a ratio based on the rate or incidence of counts.

**Table 3:** Parameter estimates with 95% confidence interval (in parentheses) for the association of mosquito density (per trap/house) with climatic variables based on univariable Poisson regression models at different lag points in time

	**IRR**^**‡ **^**(95% CI)**			
**Climatic variables**	**Current month**	**One month lag**	**Two months lag**	**Three months lag**
Rainfall(mm)	1.550 ^∗^(1.443, 1.665)	1.567 ^∗^(1.433, 1.715)	1.407 ^∗^(1.264, 1.566)	1.226(1.127, 1.334)
Relative humidity(%)	1.095 ^∗∗^(1.069, 1.122)	1.091 ^∗∗^(1.062, 1.121)	1.060 ^∗^ (1.034, 1.085)	1.027(1.013, 1.041)
Minimum temperature(^*o*^C)	1.525 ^∗∗^(1.320, 1.761)	1.900 ^∗∗^(1.454, 2.474)	2.138 ^∗∗^(1.599, 2.860)	1.853 ^∗∗^(1.494, 2.298)
Maximum temperature(^*o*^C)	0.580 ^∗^(0.474, 0.709)	0.644 ^∗^(0.527, 0.788)	0.788 ^∗^(0.635, 0.977)	1.033(0.940, 1.135)

^∗^Significant at *P* < 0.05, ^∗∗^ Significant at *P* < 0.01.

^*†*^Incidence Rate Ratio (IRR): is obtained by exponentiating the Poisson regression coefficient and it is a ratio based on the rate or incidence of counts.

**Figure 3:**
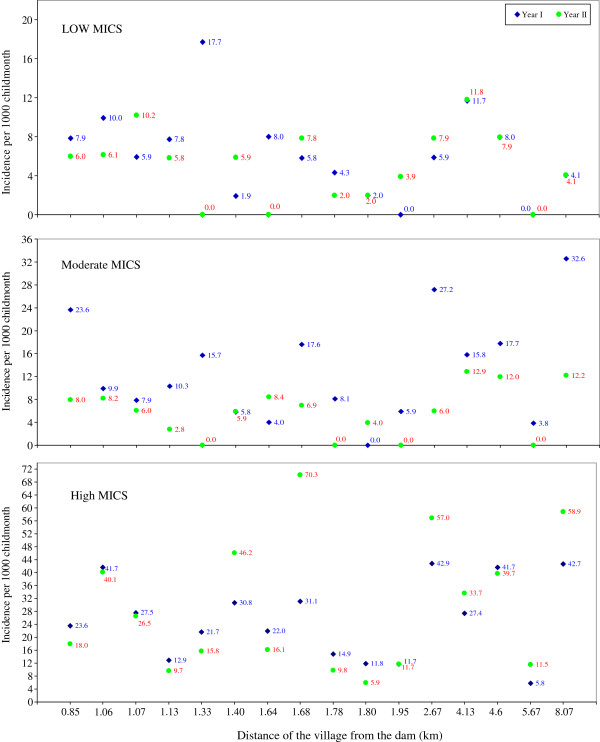
Malaria incidence (number of cases per 1000 child months) among villages located at different distance from the dam reservoir shore in different seasons (LOW: from December to March, MODERATE: from April to July and HIGH: from August to November MICS) over two years study period in Gilgel-Gibe dam area, Ethiopia.

The spatio-temporal dynamics of *P. falciparum* malaria incidence is shown in Figure 3. Over the two years follow up, the heterogeneity in malaria incidence rates between the villages was high (72.83%) compared to heterogeneity within village (27.17%).

## Discussion

Malaria transmission in the study setting occurs throughout the year but it has a seasonal pattern with an incidence peaking during October/November and with more than 75% of the cases occurring in six months. The seasonality of malaria incidence was consistent over the two years study period and is comparable to observations in other parts of the country [20].

No association between the *P. falciparum* malaria incidence and the distance from the Gilgel-Gibe dam reservoir was found. This finding was in consistent with the often stated hypothesis that dams influence malaria incidence and/or prolong the seasonality of malaria transmission by increasing mosquito abundance. Other studies from the Rift Valley of Ethiopia [21,22] indicated that dams are associated with an increased malaria risk. Other small dams constructed for irrigation in Ethiopia were also shown to be associated with higher malaria incidence [23,24].

Higher densities of the major local malaria vector, *Anopheles arabiensis*, were recorded during the wet season in villages nearer to the dam reservoir. This could be explained by the larger number of temporary mosquito breeding sites due to the presence of the reservoir.

Although higher malaria vector abundance is associated with higher malaria incidence, and mosquito abundance is higher close to the dam, no effect of the distance to the dam is found on malaria incidence. This apparent paradox can be explained by the large seasonal effects. The effect of mosquito density on malaria incidence in the univariable model disappears when controlling for season in the multivariable model. There is indeed a large difference between mosquito density and malaria incidence between the rainy and dry season. In the rainy season, mosquito density is high everywhere, and the higher mosquito number near to the dam is therefore superfluous.

Several studies also reported that only small populations of mosquitoes are required to maintain a high level of transmission [25,26]. During the dry season, the overall level of mosquitoes was low and likewise the dam did not result in a sufficient augmentation of malaria incidence.

Climate seems to be the most important determinant of malaria risk through its influence on mosquito density [27,28], as is reported elsewhere [29]. Loha and Lindtjorn [30] reported that monthly rainfall, minimum and maximum temperatures were predictors of *P. falciparum* malaria incidence in different localities in Ethiopia. In our study, minimum temperature was correlated with higher mosquito density, while maximum temperature showed no effect on mosquito density. Minimum temperature predicts malaria risk mainly due to its direct effect on the survival and feeding frequency of malaria vectors [31] and by shortening the incubation period of the parasite in mosquitoes [32]. In Uganda, average minimum temperature was also significantly associated with the number of *An. gambiae* s.l. [33]. At high altitudes, minimum temperature enhance the survival of both the parasite and the vector and thus accelerate the transmission dynamics of malaria [34]. Other similar studies conducted in different geographic regions of Ethiopia highlighted the importance of minimum temperature in predicting malaria incidence [35,36]. On the other hand, higher maximum temperature seemed to have a negative effect on mosquito density. Maximum temperature could also influence mosquito survival and may result in breeding sites drying up faster after seasonal rains in cold environments such as the current study area [37].

The contrasting effect of minimum and maximum temperature indicates that an optimal temperature might exist. Moreover, the climatic variables also showed a lag effect on malaria incidence. Rainfall was found to be a predictor of *P. falciparum* malaria incidence with 1-3 months lag period as was also observed in several other localities in Ethiopia [30].

In addition to climate, our findings indicate that the risk of malaria varied with age with older children being more at risk which is in agreement with other studies [38]. No difference was observed in malaria incidence between boys and girls.

## Conclusion

No effect of the distance of the dam on malaria incidence was found. Anthropogenic environmental changes do not necessarily result in increased mosquito-borne disease incidence in the surrounding human population as the link between anthropogenic environmental changes and disease incidence is complex and can be influenced by several other variables [39]. Hence, the relationship between malaria incidence and water resource development is often complex and local-specific [40]. It is also important to note that, as the reservoir matures, it may allow the growth of aquatic vegetation. This could provide more ideal conditions for other malaria vector species and for a vector shift that could alter the malaria transmission pattern in the area. This suggests the need for appropriate and continued vector surveillance and monitoring operations [7].

## Competing interests

The authors declare that they have no competing interests.

## Authors’ contributions

DY conceived and designed the study, was involved in the coordination and supervision and drafted the manuscript; YG performed the data cleaning, statistical analysis and critically reviewed the manuscript; KT was involved in the coordination, supervision and reviewed the manuscript; KW was involved in the study design, supervision, coordination and data entry; WK was involved in the study design, supervision, coordination and data entry; LD was involved in statistical analysis and critically reviewed the manuscript; NS was involved the statistical analysis, in drafting and critically reviewing the manuscript. All authors read and approved the final manuscript.

## Pre-publication history

The pre-publication history for this paper can be accessed here:

http://www.biomedcentral.com/1471-2334/13/161/prepub
